# Comparison of visual performance between diffractive bifocal and diffractive trifocal intraocular lenses

**DOI:** 10.1038/s41598-024-55926-5

**Published:** 2024-03-04

**Authors:** Junya Kawamura, Hirotaka Tanabe, Tomohiro Shojo, Tomofusa Yamauchi, Kosuke Takase, Hitoshi Tabuchi

**Affiliations:** 1 Department of Ophthalmology, Tsukazaki Hospital, Himeji, Japan; 2 Department of Technology and Design Thinking for Medicine, Hiroshima University Graduate School of Biomedical and Health Sciences, Hiroshima, Japan

**Keywords:** Diseases, Eye diseases, Lens diseases

## Abstract

To evaluate the visual performance of a diffractive bifocal intraocular lens (IOL) with + 4.0 D near addition (ZMB00) and a diffractive trifocal IOL with + 2.17 D and + 3.25 D near addition (AcrySof IQ PanOptix TFNT00), we investigated the 10-week postoperative parameters after cataract surgery in which ZMB00 or TFNT00 lenses were implanted bilaterally from 2011 to 2020 (with a 3-month interval between implantation of the right and left lenses). The study included 1448 eyes of 724 patients. The diffractive bifocal group comprised 1326 eyes of 663 patients (aged 67.0 ± 7.8 years; females/males, 518/145), and the diffractive trifocal group comprised 122 eyes of 61 patients (aged 66.6 ± 7.3 years; females/males, 35/26). A linear mixed-effects model using data for both eyes, with strict adjustments for sex, age, subjective refraction spherical equivalent, subjective refraction cylinder, corneal astigmatism, axial length, corneal higher-order aberrations, and pupil diameter, ensured statistical validity. Uncorrected near visual acuity and higher-order aberrations (ocular/internal, scaled to a pupil size of 4 mm) (Wavefront_4mm_postoperative_Ocular/Internal_Spherical) were significantly better in the bifocal group (*p* < 0.00068, Wald test). Uncorrected intermediate visual acuity, contrast sensitivity (6.3/4.0/2.5/1.6/1.0/0.7 degrees), and contrast sensitivity with glare (4.0/1.6/1.0/0.7 degrees) were significantly better in the trifocal group (*p* < 0.00068, Wald test).

## Introduction

Multifocal intraocular lenses (IOLs) aim to improve visual function at various distances in patients who undergo implantation. Diffractive bifocal IOLs can focus not only on distant objects but also on near objects for the purposes of reading and manual work; diffractive trifocal IOLs can also focus at an intermediate distance, mainly for tasks such as computer work and cooking. IOLs have improved patients' levels of spectacle independence and quality of life^1–6^.

The TFNT00 [Alcon Laboratories, Inc., Fort Worth, TX, USA] is a diffractive trifocal IOL that was first approved in Japan in 2019. The diffraction zone is in the center of the lens and occupies 4.5 mm of the optical zone, thus splitting the light and adding intermediate zones at approximately + 2.17 diopters (D) and + 3.25 D. The 4.5 mm diffraction zone reduces dependence on pupil size^4,7^. As the first diffractive trifocal IOL approved in Japan, it has been inserted in many patients since its introduction.

The ZMB00 [Johnson & Johnson Surgical Vision, Inc., Santa Ana, CA, USA] is a diffractive bifocal IOL that was approved in 2011. It is a single-piece bifocal hydrophobic acrylic lens that has a posterior diffractive surface and an aspheric front surface with − 0.27 μm of spherical aberration addition to the normal human eye and achieves a 4 D addition equivalent to + 3.2 D in the corneal plane^8–12^. The diffractive surface is not dependent on the pupil diameter because it includes the entire surface of the optical portion, which measures 6 mm. The ZMB00 was an early introduction to multifocal lenses in Japan, and it is a commonly used lens in the Japanese population.

Multifocal IOLs cause halo and glare and are associated with contrast sensitivity problems^13–17^. In general, it is said that contrast sensitivity decreases as the number of focal points increases in an IOL, but technological innovations have made significant improvements in this area. The ZMB00 and TFNT00 have been compared with monofocal and multifocal IOLs, including diffractive bifocal and diffractive trifocal IOLs, in several comparative trials and various discussions.

In this study, we performed a comparative analysis of the visual performance between the ZMB00, which has been implanted in a relatively large number of patients, and the TFNT00, a recently approved trifocal IOL that has entered clinical use in Japan. We believe these two IOLs are worth comparing because they have been representatives of diffractive bifocal and diffractive trifocal IOLs, respectively, in Japan.

## Results

### Patient characteristics

The patient demographics and pre/postoperative visual parameters are shown in Supplementary Table S1. The study included 1448 eyes of 724 patients. The bifocal group comprised 1326 eyes of 663 patients (aged 67.0 ± 7.8 years; females/males, 518 [78.1%]/145 [21.9%]), and the trifocal group comprised 122 eyes of 61 patients (aged 66.6 ± 7.3 years; females/males, 35 [57.4%]/26 [42.6%].)

### Comparison of postoperative parameters between Tecnis diffractive bifocal IOLs (ZMB00) and AcrySof IQ PanOptix diffractive trifocal IOLs (TFNT00)

**Figure 1:**
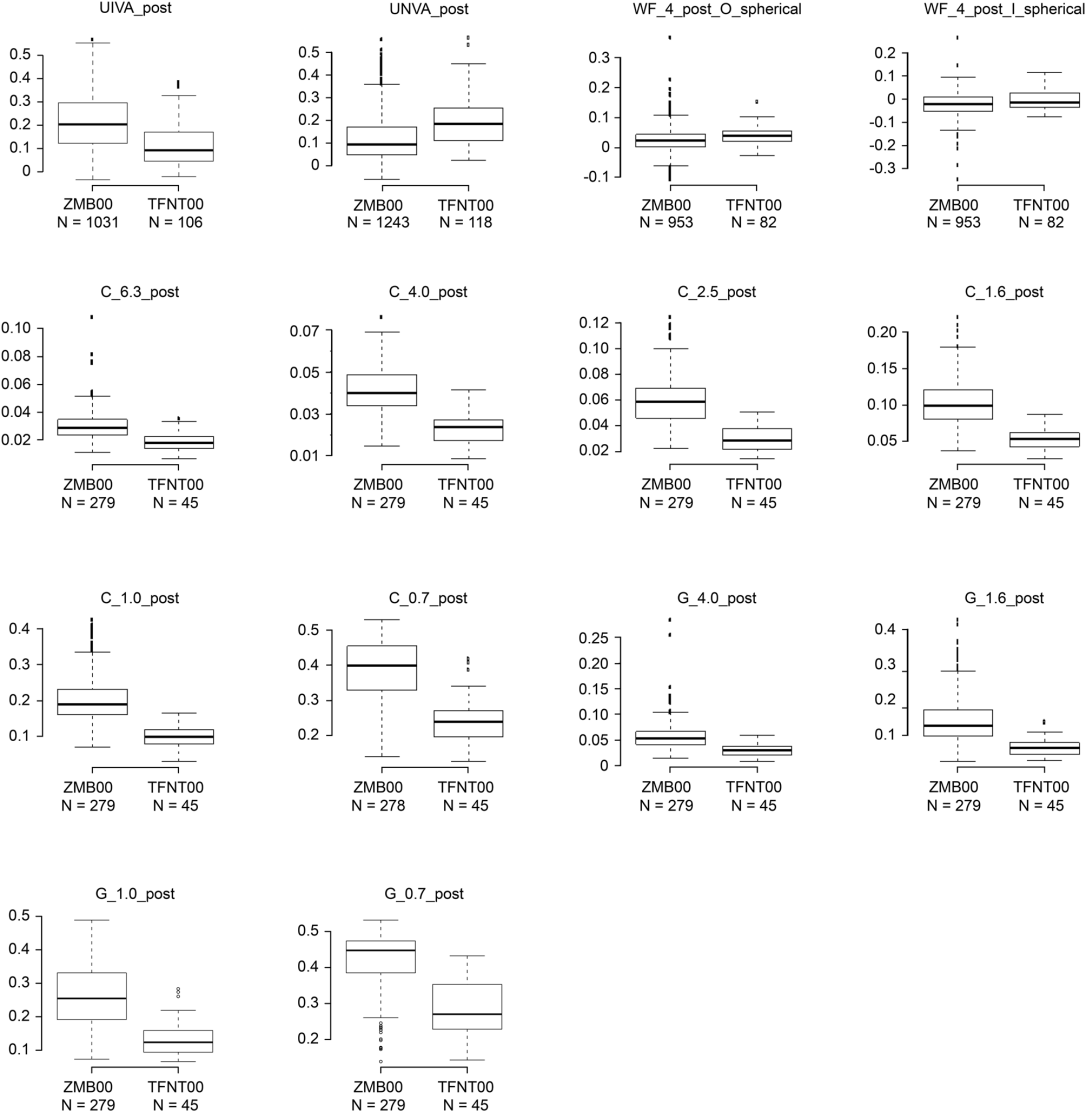
Parameters that demonstrated a significant difference between the diffractive bifocal and diffractive trifocal groups at 10 weeks after bilateral implantation was completed. The band inside the box represents the median. To highlight suspected outliers, the upper whisker is set as the maximum or the third quartile + 1.5 × IQR. The lower whisker indicates the minimum or the first quartile-1.5 × IQR. Each parameter was adjusted by multiple regression with the explanatory variables in Table 1. The Wald test (two-sided) was applied to evaluate the significance of differences between the two groups, and the significance level was set to 0.00068 using Bonferroni’s correction. The asterisk * in this figure indicates a significant difference between the two groups satisfied with the significance level of *p* < 0.00068.

**Figure 2:**
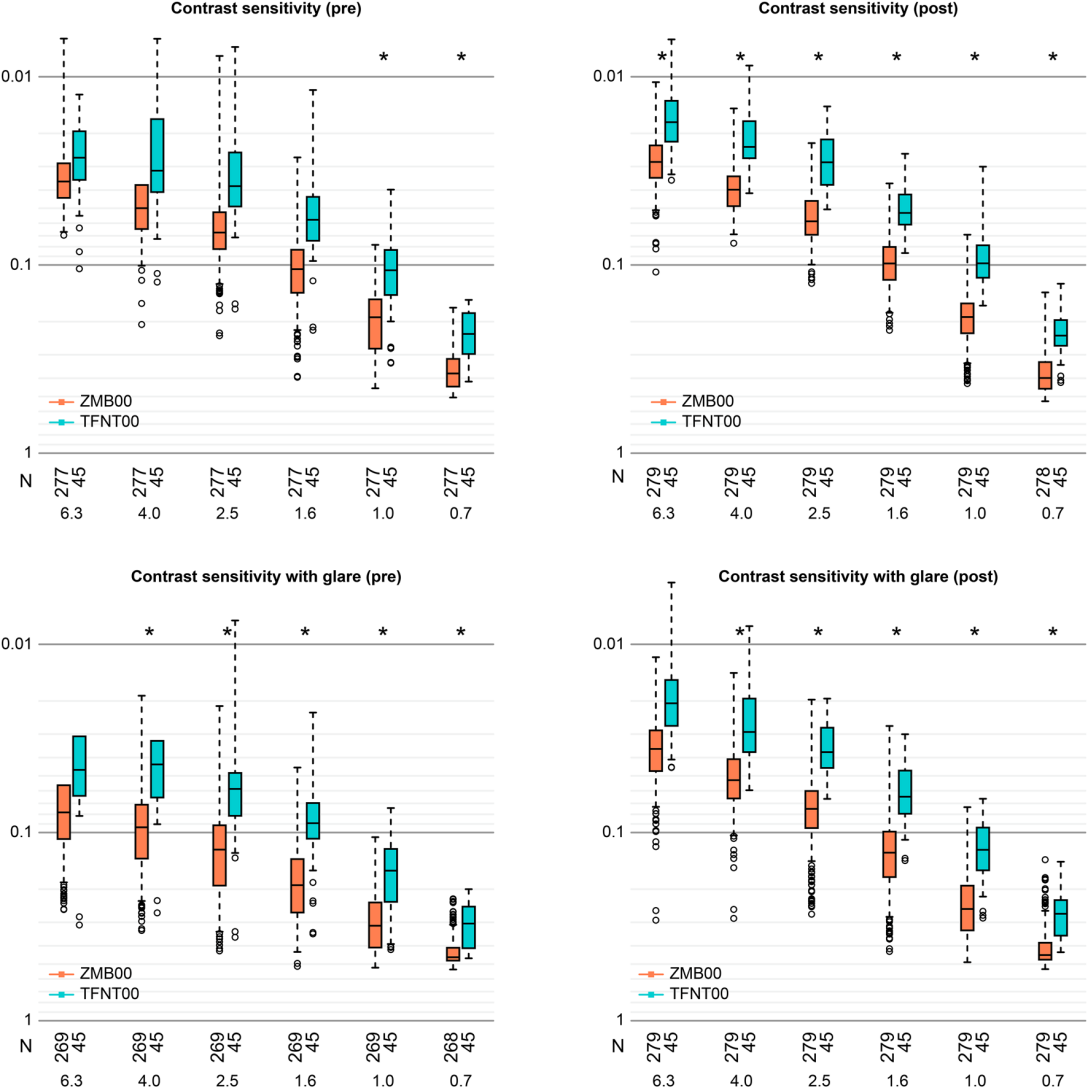
Contrast sensitivity with or without glare in the diffractive bifocal and diffractive trifocal groups before and 10 weeks after bilateral implantation was completed. In the box-and-whisker plots, the bottom of the box indicates the first quartile, and the top of the box indicates the third quartile. The band across the box represents the median. To highlight suspected outliers, the upper whisker indicates the maximum or the third quartile + 1.5 × IQR, whichever is lower. The lower whisker indicates the minimum or the first quartile—1.5 × IQR, whichever is higher. Each parameter was adjusted by multiple linear regression with the explanatory variables in Table 1. The Wald test (two-sided) was applied to evaluate the significance of differences between the two groups, and the significance level was set to 0.0083 after Bonferroni’s correction.

**Figure 3:**
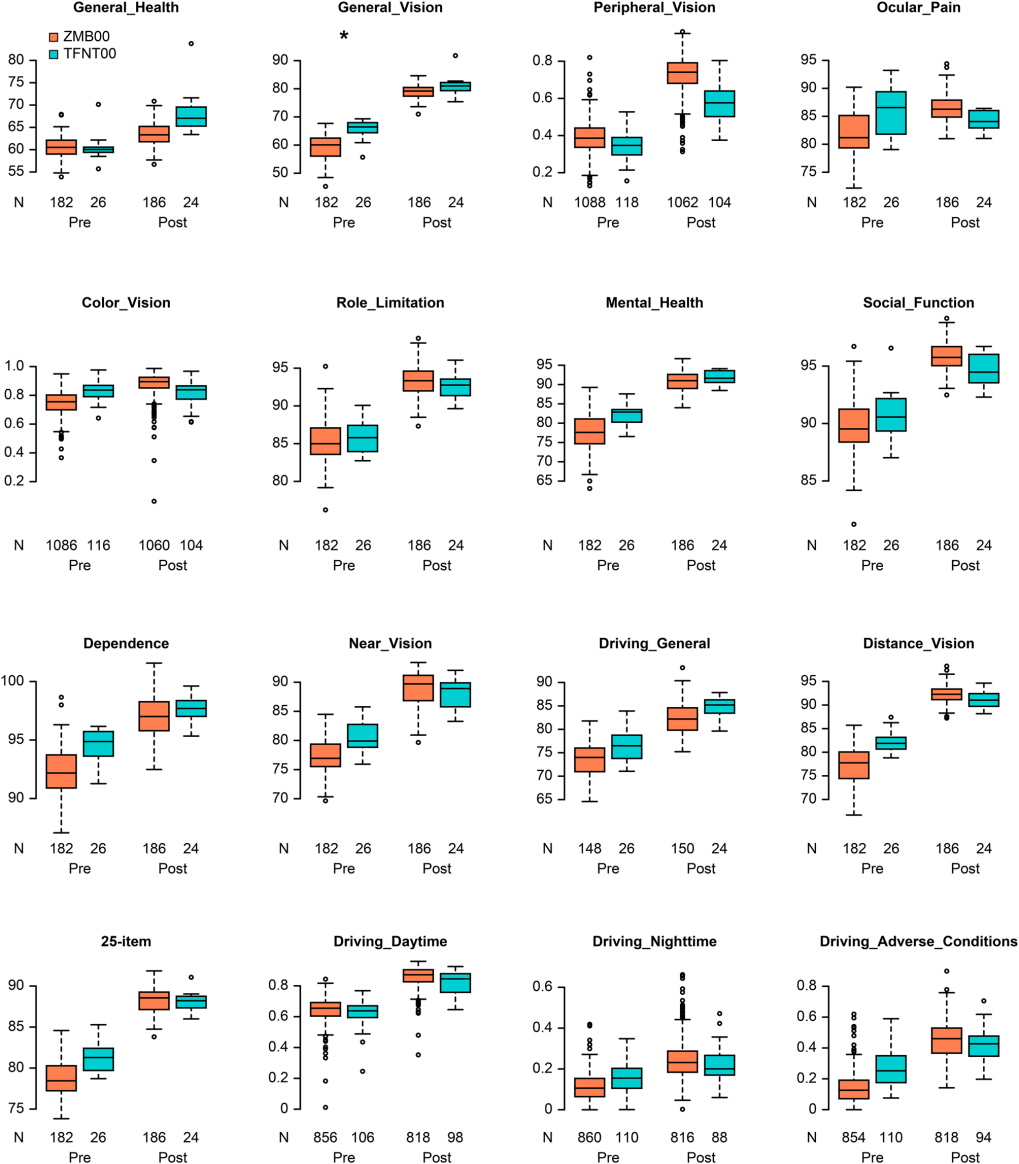
VFQ-25 scores in the diffractive bifocal and diffractive trifocal groups before/10 weeks after surgery. In the box-and-whisker plots, the bottom of the box indicates the first quartile, and the top of the box indicates the third quartile. The band across the box represents the median. To highlight suspected outliers, the upper whisker indicates the maximum or the third quartile + 1.5 × IQR, whichever is lower. The lower whisker indicates the minimum or the first quartile—1.5 × IQR, whichever is higher. Each parameter was adjusted by multiple linear regression with the explanatory variables in Table 1. The Wald test (two-sided) was applied to evaluate the significance of differences between the two groups, and the significance level was set to 0.003125 after Bonferroni’s correction. The asterisk * in this figure indicates a significant difference between the two groups satisfied with the significance level of *p* < 0.003125.

Multiple regression analysis was applied to all postoperative parameters of the diffractive bifocal and diffractive trifocal groups at 10 weeks after surgery on both eyes in the same way described in our previous study^11,12^; the parameters were adjusted by multiple regression with the explanatory variables, as presented in Table 1, and the results of the analysis are shown in Supplementary Table S2. Uncorrected near visual acuity and higher-order aberrations (ocular/internal, scaled to a pupil size of 4 mm) (Wavefront [WF]_4 mm^4^ postoperative [post]_Ocular [O]/Internal [I]_Spherical) were significantly better in the diffractive bifocal group (*p* < 0.00068, Wald test), and corrected distance visual acuity was slightly but significantly better in the diffractive bifocal group (*p* < 0.05, Wald test) (Table 2 and Fig. 1). Uncorrected intermediate visual acuity, contrast sensitivity (6.3/4.0/2.5/1.6/1.0/0.7 degrees), and contrast sensitivity with glare (4.0/1.6/1.0/0.7 degrees) were significantly better in the diffractive trifocal group (*p* < 0.00068, Wald test), and contrast sensitivity with glare (6.3/2.5 degrees) was slightly but significantly better in the diffractive trifocal group (*p* < 0.05, Wald test) (Table 2, Figs. 1 and 2). Although National Eye Institute (NEI) Visual Function Questionnaire (VFQ)-25 scores for Social_Function, Distance_Vision and Peripheral_Vision were slightly but significantly better in the diffractive bifocal group (*p* < 0.05, Wald test) (Table 2 and Fig. 1), VFQ-25 scores in the diffractive bifocal and diffractive trifocal groups were not significantly different at the significance level of *p* < 0.00068, Wald test or *p* < 0.003125, Wald test (Table 2 and Fig. 3).

**Table 1:** Parameters in the diffractive bifocal and diffractive trifocal groups used to adjust the linear regression model: age, sex, axial length (at the time of surgery), subjective refraction spherical equivalent (SE), subjective refraction cylinder (CYL), corneal astigmatism (keratometric cylinder), corneal higher-order aberrations (astigmatism, total higher-order aberration (HOA), third, fourth, trefoil, coma, tetrafoil, second-order astigmatism (2ndAstig), and spherical, scaled to a pupil size of 4 mm/6 mm), and pupil diameter.

(A) Categorical variable				
		N (%)		
Variable	Levels	ZMB00	TFNT00	*p* value (Wald test)
Sex	F/M	518 (78.1)/145 (21.9)	35 (57.4)/26 (42.6)	4.754E−04**

(B) Continuous variables				
		N, Mean ± SD		
Variable		ZMB00	TFNT00	*p* value (Wald test)
Age		663, 67.043 ± 7.809	61, 66.590 ± 7.251	3.248E−01
SE		1069, 0.229 ± 0.448	98, 0.000 ± 0.405	1.144E−06**
CYL		849, − 0.785 ± 0.397	67, − 0.612 ± 0.243	1.013E−03**
Corneal Astigmatism		525, − 0.731 ± 0.413	115, − 0.583 ± 0.486	1.288E−06**
Axial Length		1326, 24.045 ± 1.574	122, 24.191 ± 1.390	9.756E−02
WF_4_post_C				
Astigmatism		953, − 0.873 ± 0.501	82, − 0.623 ± 0.329	9.241E−06**
Total HOA		953, 0.204 ± 0.105	82, 0.205 ± 0.130	6.673E−01
Third		953, 0.174 ± 0.100	82, 0.183 ± 0.131	7.430E−01
Fourth		953, 0.098 ± 0.054	82, 0.081 ± 0.040	6.917E−05**
Trefoil		953, 0.131 ± 0.086	82, 0.129 ± 0.114	2.955E−01
Coma		953, 0.100 ± 0.075	82, 0.117 ± 0.085	2.122E−02*
Tetrafoil		953, 0.059 ± 0.045	82, 0.043 ± 0.033	7.535E−05**
2nd Astig		953, 0.039 ± 0.030	82, 0.035 ± 0.029	7.608E−02
Spherical		953, 0.048 ± 0.047	82, 0.042 ± 0.037	3.646E−01
WF_6_post_C				
Astigmatism		868, − 0.623 ± 0.433	79, − 0.396 ± 0.238	1.623E−07**
Total HOA		868, 0.580 ± 0.435	79, 0.494 ± 0.181	7.982E−04**
Third		868, 0.390 ± 0.310	79, 0.358 ± 0.192	3.196E−01
Fourth		868, 0.368 ± 0.249	79, 0.293 ± 0.093	2.355E−07**
Trefoil		868, 0.278 ± 0.236	79, 0.232 ± 0.179	9.642E−03*
Coma		868, 0.245 ± 0.235	79, 0.249 ± 0.128	9.632E−02
Tetrafoil		868, 0.169 ± 0.180	79, 0.088 ± 0.062	6.485E−12**
2nd Astig		868, 0.100 ± 0.158	79, 0.078 ± 0.049	3.074E−01
Spherical		868, 0.274 ± 0.162	79, 0.233 ± 0.143	7.232E−03*
Pupil Diameter post		980, 4.480 ± 0.871	82, 4.715 ± 0.777	5.829E−03*

For categorical data, each category and its count and frequency are shown, and Fisher’s exact (two-sided) test was used to compare categorical data for the diffractive bifocal and diffractive trifocal IOLs. For numerical data, the mean and standard deviation are shown, and the Mann–Whitney U test (two-sided) was used to compare numerical data for the bifocal and trifocal IOLs.

*p < 0.05, **p < 0.002 (= 0.05/25).

SE: subjective refraction spherical equivalent; CYL: subjective refraction cylinder; WF_4_post_C_: wavefront_4_post_corneal; HOA: higher-order aberration.

**Table 2:** Parameters that demonstrated a significant difference at *p* < 0.00068 or *p* < 0.05 between the diffractive bifocal and diffractive trifocal groups at 10 weeks after bilateral implantation was completed.

Response_post	After adjustment			
	ZMB00	TFNT00	Coefficient (95% CI)	*p* value (Wald test)
UIVA	0.22 ± 0.12	0.11 ± 0.09	− 0.10(− 0.15,− 0.06)	4.102E−06**
UNVA	0.12 ± 0.09	0.19 ± 0.11	0.10(0.07,0.13)	2.702E−09**
WF_4_post_O				
Spherical	0.02 ± 0.04	0.04 ± 0.03	0.03(0.02,0.04)	3.099E−06**
WF_4_post_I				
Spherical	− 0.03 ± 0.04	− 0.01 ± 0.04	0.03(0.02,0.04)	3.099E−06**
Contrast sensitivity				
C_6.3	0.03 ± 0.01	0.02 ± 0.01	− 0.01(− 0.01,− 0.00)	2.945E−04**
C_4.0	0.04 ± 0.01	0.02 ± 0.01	− 0.01(− 0.02,− 0.01)	1.309E−10**
C_2.5	0.05 ± 0.01	0.03 ± 0.01	− 0.02(− 0.03,− 0.01)	3.946E−11**
C_1.6	0.09 ± 0.03	0.06 ± 0.03	− 0.03(− 0.04,− 0.02)	1.269E−06**
C_1.0	0.18 ± 0.06	0.12 ± 0.06	− 0.06(− 0.09,− 0.04)	7.712E−08**
C_0.7	0.36 ± 0.07	0.25 ± 0.07	− 0.10(− 0.13,− 0.07)	4.250E−11**
Contrast sensitivity with glare				
G_6.3	0.03 ± 0.02	0.02 ± 0.01	− 0.01(− 0.02,− 0.00)	7.023E−03*
G_4.0	0.05 ± 0.02	0.03 ± 0.01	− 0.01(− 0.02,− 0.01)	1.714E−04**
G_2.5	0.07 ± 0.03	0.05 ± 0.03	− 0.02(− 0.03,− 0.01)	8.185E−04*
G_1.6	0.12 ± 0.05	0.08 ± 0.04	− 0.03(− 0.05,− 0.02)	2.636E−04**
G_1.0	0.22 ± 0.08	0.15 ± 0.07	− 0.07(− 0.10,− 0.04)	5.072E−06**
G_0.7	0.39 ± 0.07	0.31 ± 0.08	− 0.09(− 0.12,− 0.06)	7.724E−09**
VFQ-25				
Social_Function	95.95 ± 1.34	94.86 ± 1.88	− 2.11(− 3.75,− 0.47)	1.164E−02*
Distance_Vision	92.35 ± 2.29	91.49 ± 2.37	− 2.27(− 4.32,− 0.22)	2.976E−02*
Peripheral_Vision (low/high)	121/410	43/9	− 0.70(− 1.16,− 0.24)	3.134E−03*

Each parameter was adjusted by multiple regression with the explanatory variables in Table 1. For each response variable, the mean and standard deviation for each numerical parameter or the counts for each categorical parameter (Spectacle Dependence: never/sometimes/always), the regression coefficient, its 95% confidence interval, and the *p* value (Wald test) are shown.

*p < 0.05, **p < 0.00068.

UIVA: uncorrected intermediate visual acuity; UNVA: uncorrected near visual acuity; WF_4_post_O_: wavefront_4_post_ocular; WF_4_post_I_: wavefront_4_post_internal; C: contrast sensitivity; G: contrast sensitivity under glare.

### Correlation among postoperative parameters of Tecnis diffractive bifocal IOLs (ZMB00) and AcrySof IQ PanOptix diffractive trifocal IOLs (TFNT00)

**Figure 4:**
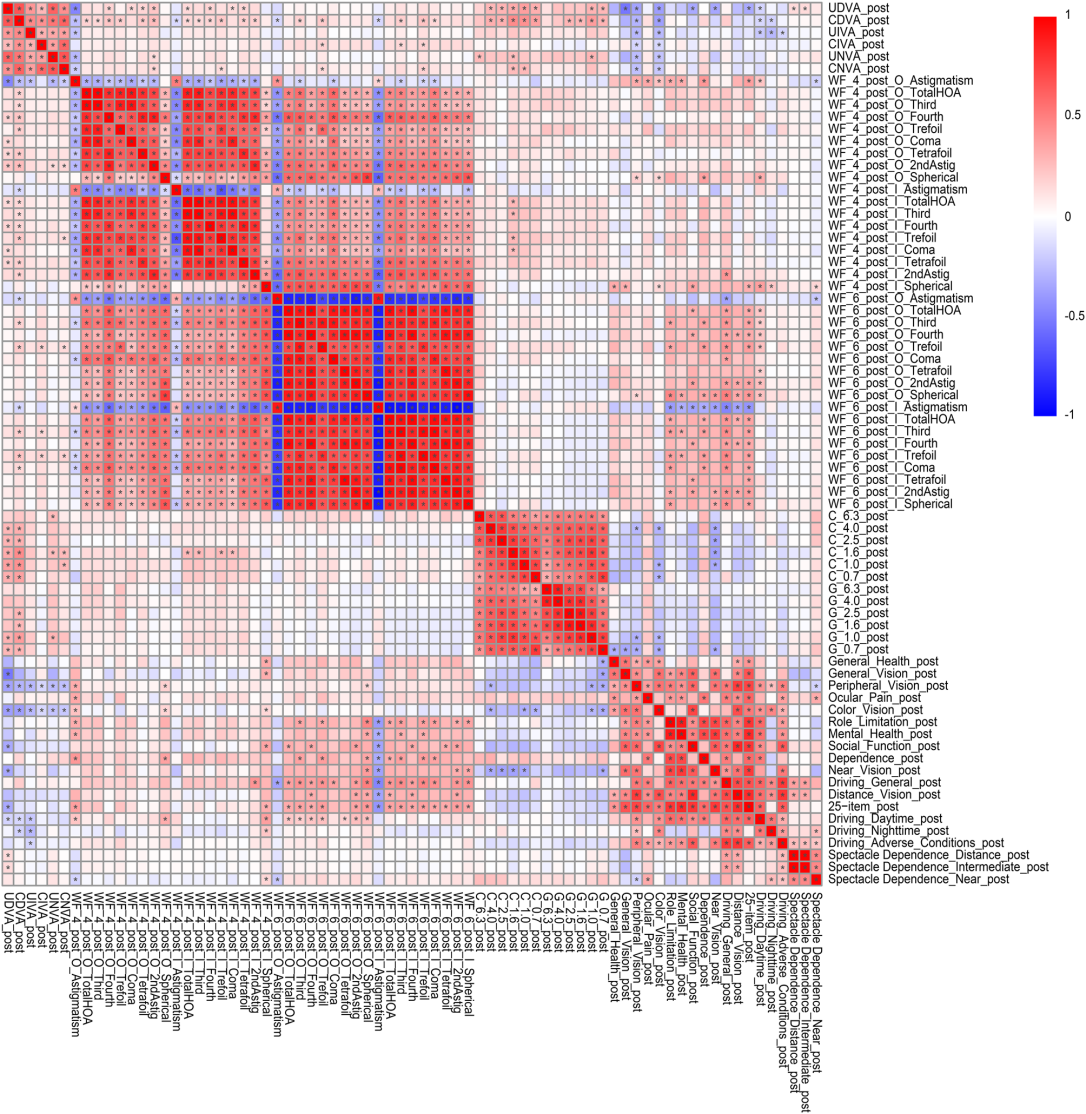
Heatmap of Pearson’s correlation coefficients between all possible combinations of variables, which were adjusted by multiple regression with the explanatory variables in Table 1, in the diffractive bifocal IOL group. The asterisk * in this figure indicates a significant correlation between two parameters at *p* < 0.00002 after Bonferroni’s correction. The t test (two-sided) was applied to evaluate the significance of differences between the two groups. The sample size for each parameter is shown in Supplementary Table 3(C). The illustration was performed using a commercially available software program (R, version 3.6.1; R Core Team, 2019, Vienna, Austria.)^38^ (https://cran.r-project.org/web/packages/pheatmap/pheatmap.pdf).

**Figure 5:**
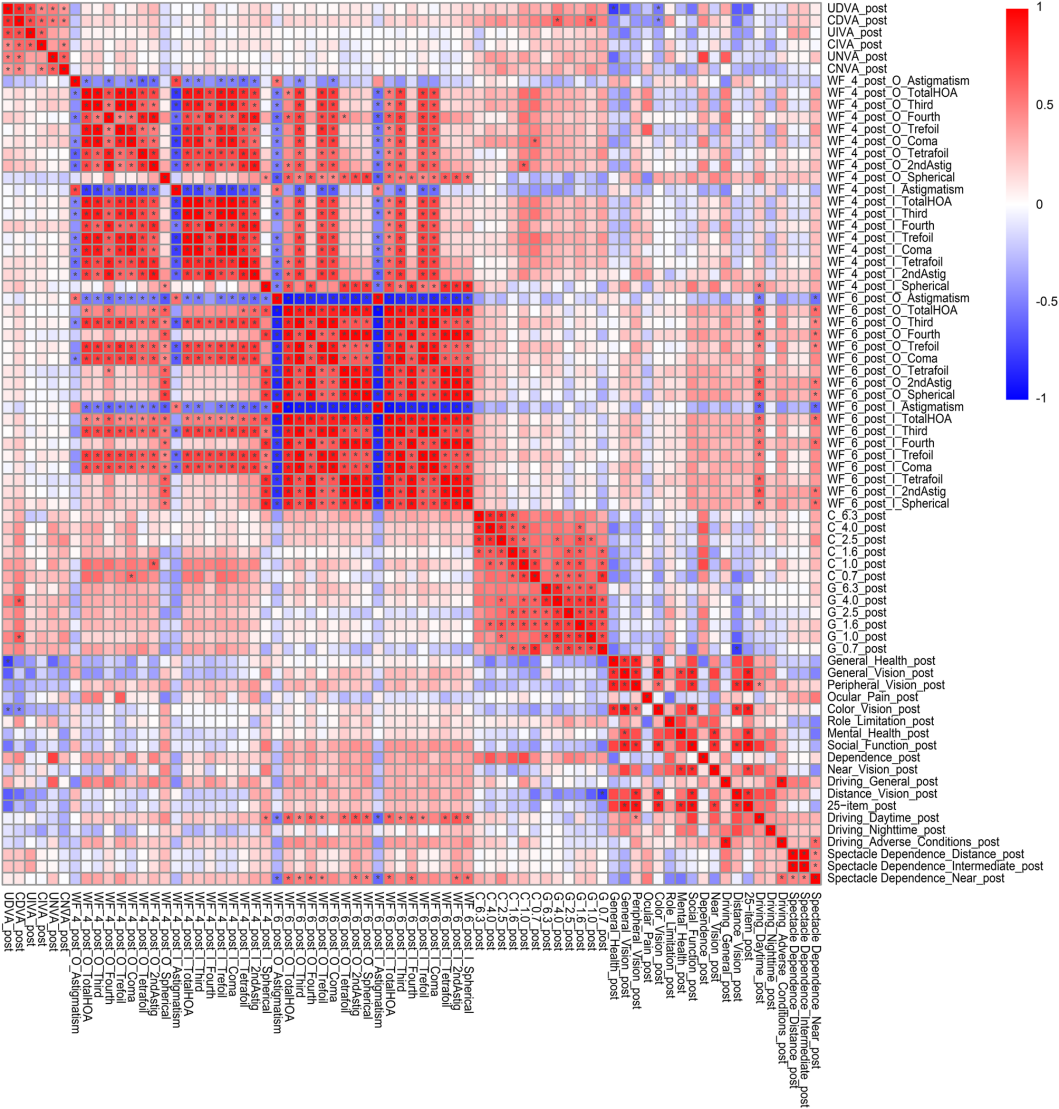
Heatmap of Pearson’s correlation coefficients between all possible combinations of variables, which were adjusted by multiple regression with the explanatory variables in Table 1, in the diffractive trifocal group. The asterisk * in this figure indicates a significant correlation between two parameters at *p* < 0.00002 after Bonferroni’s correction. The t test (two-sided) was applied to evaluate the significance of the differences between the two groups. The sample size for each parameter is shown in Supplementary Table 4(C). The illustration was performed using a commercially available software program (R, version 3.6.1; R Core Team, 2019, Vienna, Austria)^38^ (https://cran.r-project.org/web/packages/pheatmap/pheatmap.pdf).

The correlation coefficients (A) and *p* values for the correlation analyses (B) between all possible combinations of postoperative parameters for the diffractive bifocal and diffractive trifocal groups were adjusted by multiple regression with the explanatory variables in Table 1 as described in our previous study^11,12^. The results of each lens are shown in Supplementary Table S3 and Fig. 4 and Supplementary Table S4 and Fig. 5, respectively.

## Discussion

Diffractive bifocal IOLs divide light into two foci; in Tecnis multifocal IOLs, 41% of incoming light is used for distance vision and 41% for near vision, independent of the pupil diameter, whereas the remaining 18% is lost to higher-order scattering^18,19^. Diffractive trifocal IOLs divide light into three foci; moreover, PanOptix lenses are relatively unaffected regardless of pupil diameter, with 88% of the incoming light reaching the retina. 50% of this is used for distance vision, 25% for intermediate vision and 25% for near vision^7^. Cochener^20^ evaluated the postoperative visual function of bifocal IOL (ZMB00) and trifocal IOL (FineVision) and reported a better visual acuity with the trifocal IOL for intermediate vision and no difference for near vision. In our study, intermediate visual acuity was better with trifocal IOL than with bifocal IOL, but near visual acuity was better with bifocal IOL than with trifocal IOL. Tecnis multifocal IOLs have a prescription power of + 4 D and an ideal near focal length of 33 cm, and PanOptix lenses have a prescription power of + 2.17 D and + 3.25 D, with ideal focal distances of 40 cm in the near range and 60 cm in the intermediate range. The far vision was tested at 5 m, the intermediate vision was tested at 50 cm, and the near vision was tested at 30 cm for the examination of visual acuity in our study, and Tecnis multifocal IOLs had significantly better near visual acuity than PanOptix, which was partially due to the fact that their ideal near focal length is closer to the near acuity test distance. A greater percentage of spectral proportions in the near region of Tecnis multifocal IOLs may have also contributed to the better near visual acuity.

Approximately 82.2% of patients in the bifocal group and 83.7% of patients in the trifocal group were completely spectacle independent. This is consistent with the findings of other study reports on Tecnis multifocal IOLs and PanOptix IOLs, in which the percentage ranges from 82.6 to 92.8%^21–25^ and from 85.0 to 96.3%^26,27^, respectively. In this study, although the results showed that the TFNT00 was significantly superior to the ZMB00 in terms of intermediate visual acuity and that ZMB00 was significantly superior to the TFNT00 in terms of near visual acuity, there was no significant difference in spectacle independence. Good intermediate vision is necessary when using digital devices, such as computers, smartphones, and tablets, and when cooking and playing sports. Unlike the TFNT00, which is a diffractive trifocal IOL, the ZMB00, a diffractive bifocal intraocular lens, does not have spectral distribution at intermediate distances; nevertheless, there are reports that the ZMB00 provides functional intermediate vision^6^. Moreover, Japanese individuals, who have high percentages of myopia and presbyopia and are likely to need eyeglasses or contact lenses, can expect an improved quality of vision with the spectacle independence that they gain after TFNT00/ZMB00 insertion.

We used the CGT-1000 instrument to measure contrast sensitivity. The CGT-1000 is a dome-shaped device that measures contrast sensitivity by converting the size and luminance of the optotype, which is indicated by a double ring displayed on the monitor at a constant background luminance of 10 cd/m^2^ and an examination distance of 35 cm. Patients undergoing this examination are asked if they can detect any changes in the brightness contrast of a circular optotype of variable size consisting of three colored concentric circles. This device can evaluate contrast sensitivity at six sizes and 13 contrast levels, with/without glare^28^. Jonker et al.^29^ reported that the mean mesopic contrast sensitivity was higher in the bifocal group (Acrysof IQ Restor + 3.0 bifocal IOLs) than in the trifocal group (Finevision Micro F trifocal IOLs). In contrast, Plaza-Puche et al.^30^ reported that there was no significant difference in the low mesopic contrast sensitivity function among various groups, including the bifocal groups (Lentis Mplus-LS313, Acri.Lisa 366D, Acrysof ReSTOR SN6AD1) and trifocal groups (AT LISA tri 839MP, FineVision). Mojzis et al.^31^ also reported that there was no significant difference in contrast sensitivity between the bifocal group (AT LISA 801) and the trifocal group (AT LISA tri 839 MP) for most of the analyzed frequencies, and Bilbao-Calabuig et al.^32^ reported that there was no significant difference in contrast sensitivity between the bifocal group (ReSTOR + 2.50 and + 3.00 D) and trifocal group (FineVision). However, García-Pérez et al.^4^ reported that TFNT00 had slightly better contrast sensitivity compared to other trifocal IOLs, FineVision and ATLISA, and Cochener^20^ reported that there was no significant difference between the ZMB00 and FineVision. In our study, the result that the TFNT00 had better contrast sensitivity than the ZMB00 is consistent with the findings of these reports. In this study, contrast sensitivity was measured under corrected near vision. It can be said that the test was performed under distance optics, which produces higher visual acuity when near vision is corrected. Although TFNT00 is a quadrifocal IOL in lens design, it functions as a trifocal IOL. There is a focal length of 120 cm in addition to 40 cm and 60 cm, and this focus (120 cm) is actually redistributed to the distant focal point to enhance visual function at a distance (Enlighten optical technology)^2,7^. This technology allows the TFNT00 to achieve the aforementioned spectral distribution of incident light to the far end of the spectrum of 44%, which is higher than the 41% of the ZMB00. This might be one reason that the contrast sensitivity (with/without glare) of the TFNT00 was better than that of the ZMB00 in this study.

The correlation coefficients between all possible combinations of variables described in the heatmaps demonstrated that most contrast sensitivity (with/without glare) of most frequencies and UDVA/CDVA were strongly correlated in the diffractive bifocal group and that contrast sensitivity with glare (4.0/1.0 degrees) and CDVA were strongly correlated in the diffractive trifocal group (Figs. 4 and 5). In other words, CDVA is related to contrast sensitivity with glare in both the TFNT00 and ZMB00 lenses.

The NEI VFQ-25, which evaluates 25 items across 12 subscales, is one of the representative multidimensional questionnaires that have been translated into several languages and assesses the general quality of life relating to eye conditions/visual problems^33^. Suzukamo et al*.*^34^ validated the Japanese version of the NEI VFQ-25*.* The heatmap of the correlation coefficients in the diffractive bifocal IOL group demonstrated that the VFQ-25 score for Peripheral Vision and Color Vision correlated with respectively visual acuity (corrected/uncorrected) and (Fig. 4). A heatmap of the correlation coefficients in the diffractive trifocal IOL group demonstrated that the VFQ-25 score for Daytime Driving and Spectacle Dependence correlated with most ocular/internal higher-order aberrations scaled to a pupil size of 6 mm and for Color Vision correlated with CDVA/UDVA (Fig. 5). Therefore, there may be a relationship between color vision and CDVA/UDVA in TFNT00/ZMB00. One of the problems experienced by patients with multifocal IOLs is night driving, and even with the TFNT00 and ZMB00, night driving tends to be less favorable than other parameters on the Visual Function Index questionnaire (VF-14) and the NEI VFQ-25.^2,6,10^. Kohnen et al.^2^ reported that 93% of patients perceived optical phenomena such as halos (89%), glare (11%), double vision (7%), ghosting (4%), and distorted vision (4%), while 7% of patients did not report any optical phenomena with TFNT00. It is known that the neural adaptation process of vision is necessary for the brain to adapt to the different images provided by multifocal optics. Failure of this neural adaptation can result in the perception of glare, confusion, distortion, and a sense of decreased visual acuity. The typical process of neural adaptation after multifocal IOL implantation takes a minimum of 3 months, with maximum improvement reached 1 year after surgery^3^. In this study, the NEI VFQ-25 was performed at 10 weeks, at which point the process of neural adaptation was probably incomplete. This could be one of the reasons for the relatively low mean night driving scores in our study population.

There are some potential limitations of this study. One of them is that intermediate visual acuity was measured only at 50 cm and near visual acuity at 30 cm in the same way described in our previous study^11,12^. Although there has been a long-standing controversy about the examination distance^35^, visual acuity should ideally be measured at various distances to gauge the performance of the lenses. As the practical distance at examination has been reported to be an important factor, we used the distances that are within arm’s reach when the patient holds a test chart; because Japanese people have relatively short average height and arm length compared with European and American people, we measured intermediate visual acuity at 50 cm, which was assumed to be within the reach of most patients in this study. However, given that the trifocal IOL was created to address the gap for intermediate vergence (V-pattern) in the delocalization curve in bifocal IOLs, which corresponds to near and far vergence, visual acuity should be measured in a wider range instead of the most representative distances to access more detailed information in this study.

Another limitation is that the preoperative status of the patient's visual performance could affect the postoperative data, especially the VFQ-25 score. However, because we evaluated the postoperative data 10 weeks after the operation, the change from the preoperative status could be considered significant to reach a stable level. This is why most studies regarding different IOL comparisons don't mention the preoperative status of visual performance. Nevertheless, potential differences in socioeconomic status might exist between the patients in the two groups in this study. To increase the validity of the study, this large-scale, single-center study was performed under a consistent protocol as described in our previous study^11,12^; i.e., after written informed consent was obtained from all the patients before surgery, we evaluated the same series of pre- and postoperative parameters, including the VFQ-25 score. Although this study was retrospective, each patient who was implanted with lenses was randomly and independently sampled, and all endpoints were measured. On the other hand, there are several postoperative differences in visual parameters, including refractions between the two groups. To address this concern, we strictly adjusted for age, sex, axial length, subjective refraction SE, subjective refraction CYL, corneal astigmatism (keratometric cylinder), corneal higher-order aberrations, and pupil diameter in the same way as described in our previous study^11,12^. Because the data contained a mixture of items evaluated either in both eyes or in each eye separately, we implemented an analysis that accounted for bias by using a linear mixed model and corrected for multiple observations for each eye per patient. It is common in statistics to assume that random assignment does not bias the results of the analysis, even if there are differences in the number of patients, such as the 1:n allocation used in clinical trials. In all multivariate analyses, the missing values of explanatory variables were filled in by the multiple imputation method with 10 imputations.

Finally, the lack of an identical platform is another limitation factor in this study. Although we compared these two IOLs because they have been representatives of diffractive bifocal IOLs and diffractive trifocal IOLs, respectively, in Japan, ideally, the platform should be consistent to perform a pure comparison of the different natures inherent in the different structures.

In conclusion, we compared the comprehensive visual performance of a diffractive trifocal IOL with a diffractive bifocal IOL, which is another multifocal lens of a similar diffractive type. Patients in the bifocal group had better uncorrected near visual acuity, whereas patients in the trifocal group had better uncorrected intermediate visual acuity and contrast sensitivity (with/without glare). At high-performance levels, the two IOL groups had different characteristics regarding various visual parameters.

## Methods

### Design

Retrospective comparative case series.

### Setting

Ophthalmology, Tsukazaki Hospital, Japan.

### Patients

We reviewed a consecutive case series of cataract patients who underwent bilateral implantation of Tecnis diffractive bifocal IOLs with + 4.0 D near addition (ZMB00) and AcrySof IQ PanOptix diffractive trifocal IOLs with + 2.17 D and + 3.25 D near addition (TFNT00) from August 11, 2011, to March 26, 2020, in the same way described in our previous study^11,12^. The right and left lenses were implanted within 3 months of each other. Participants were recruited for enrollment in this consecutive case series study (outpatients with or without doctor referral). There is no potential self-selection bias that is likely to impact the results. The exclusion criteria included a history of other ocular diseases that could affect visual function, |subjective refraction spherical equivalent (SE)|> 2.00 D, |subjective refraction cylinder (CYL)|> 3.00 D and |corneal astigmatism (keratometric cylinder)|> 3.00 D at 10 weeks after surgery.

### Preoperative examination

Preoperatively, all patients received full ophthalmologic examinations, including evaluations of the corneal curvature radius, corneal astigmatism, axial length, refractive status, ocular aberrations, pupil diameter, distance/intermediate/near visual acuity, contrast sensitivity, and contrast sensitivity under glare, as well as anterior segment evaluations using a slit lamp, tonometry, and indirect fundoscopy, in the same way described in our previous study^11,12^. The quality of vision was evaluated using the Japanese version of the NEI VFQ-25^34^. The NEI VFQ-25 was administered by experienced technicians or nurses in a face-to-face setting. Spectacle use was also evaluated by inquiring how often the patient used spectacles for distance, intermediate and near vision (with possible responses of ‘never,’ ‘sometimes’ or ‘always’).

Uncorrected distance visual acuity (UDVA) and corrected distance visual acuity (CDVA) were measured at 5.0 m. Uncorrected intermediate visual acuity (UIVA) and corrected intermediate visual acuity (CIVA) were measured at 0.5 m. Uncorrected near visual acuity (UNVA) and corrected near visual acuity (CNVA) were measured at 0.3 m. Visual acuity was measured using the decimal visual acuity chart, and the measured decimal values were converted to the logarithm of the minimum angle of resolution (logMAR) scale. The corneal curvature radius, corneal astigmatism and objective refractive status were measured using a KR-8900 autorefractor keratometer (Topcon, Tokyo, Japan). The axial length was measured using IOL Master (Carl Zeiss, Oberkochen, Germany) and AL-3000 (TOMEY, Nagoya, Japan) biometers. Contrast sensitivity and contrast sensitivity under glare were measured using a CGT-1000 contrast glare tester (Takagi Seiko, Nakano, Japan), and the pupil diameter and ocular aberrations were measured using a KR-1W Wavefront Analyzer (Topcon, Tokyo, Japan). All measurements were obtained by experienced technicians.

### IOLs and surgical technique

The patients chose to undergo implantation with either bifocal or trifocal IOLs after they had been informed of the advantages and disadvantages associated with each type in the same way as in our previous study^11,12^. Patients in the bifocal group received Tecnis diffractive bifocal IOLs with + 4.0 D near addition (ZMB00) bilaterally, while those in the diffractive trifocal group received AcrySof IQ PanOptix trifocal IOLs with + 2.17 D and + 3.25 D near addition (TFNT00) bilaterally. Both ZMB00 and TFNT00 are 1-piece aspheric hydrophobic IOLs. The ZMB00 has a modified anterior prolate surface designed to minimize spherical aberration and to improve contrast sensitivity under mesopic conditions after cataract surgery^22,36,37^. The lens also has diffractive areas on the entire 6 mm optical zone. The TFNT00 has a modified posterior prolate surface designed and a blue filter, which can be considered unique based on the principle of optical effect it uses^2,7^. The lens also has a central 4.5 mm diffraction region with 15 diffraction bands and a 6.0 mm optical band consisting of an outer refractive edge. Eleven experienced cataract surgeons performed cataract surgery using the same standard technique of sutureless microincision phacoemulsification and the same protocol. The surgical procedures consisted of topical anesthesia, the creation of a scleral or corneal incision of 1.8–2.8 mm, 5 mm of continuous capsulorhexis, phacoemulsification cataract extraction and IOL implantation with an injector.

### Postoperative examination

Patients were evaluated at 10 weeks postoperatively. The postoperative examination protocol at 10 weeks was identical to the preoperative protocol.

### Statistical analyses

The sample size was calculated for an alpha of 0.00068 and a power of 0.80. A standard deviation in VA of 0.10 logMAR units was presumed in addition to a minimum detectable difference of 1 line of VA (0.1 logMAR), which was based on our previous study^11^. This calculation recommended the inclusion of 39 eyes per group. The diffractive bifocal group comprised 1326 eyes of 663 patients, and the diffractive trifocal group comprised 122 eyes of 61 patients, respectively, and the sample size was sufficient.

As in our previous study^11,12^, the diffractive bifocal IOL and diffractive trifocal IOL groups were compared in terms of the following postoperative parameters at 10 weeks after bilateral implantation was completed: (1) mixed-effects linear regression: visual acuity (uncorrected/corrected, distance/intermediate/near), contrast sensitivity (with/without glare), and higher-order aberrations (ocular/internal, scaled to a pupil size of 4 mm/6 mm); (2) linear regression model or logistic regression: VFQ-25 score; and (3) cumulative logistic regression: spectacle dependence (distance/intermediate/near). Both groups were adjusted for age, sex, axial length, subjective refraction spherical equivalent, subjective refraction cylinder, corneal astigmatism, corneal higher-order aberrations, and pupil diameter. In the regression analyses (2) and (3), the data were divided into two parts (left-eye data and right-eye data), and the regression model was applied to each dataset. Since discrete scores were observed for "Peripheral_Vision", "Color_Vision", "Driving_Daytime", "Driving_Nighttime", "Driving_Adverse_Conditions" in VFQ-25, we treated them as binary data. We divided the patients into two groups (those with scores of 75 or lower and those with scores above 75) and applied the logistic regression model to them. The results of the left- and right-eye analyses were combined using the inverse variance method; the corrected values were calculated for the left- and right-eye datasets, and the average values were used. In all multivariate analyses, the missing values of explanatory variables were filled in by the multiple imputation method.

In the regression analysis, the Wald test was applied to evaluate the significance of differences in postoperative parameters between the two groups, and the significance level was set to 0.00068 after Bonferroni’s correction. Correlation analysis between postoperative parameters was applied for the diffractive bifocal and diffractive trifocal groups, and a heatmap of Pearson’s correlation coefficients was generated for each group. In the correlation analysis, a t-test (two-sided) was used to evaluate whether the correlation coefficient was significantly different from zero, and the significance level was set to 0.00002 after Bonferroni’s correction.

The statistical analyses were performed by using a commercially available software program (R, version 3.6.1; R Core Team, 2019, Vienna, Austria)^38^, and a multiple imputation analysis was implemented by using "mice" in the R function library^39^.

### Ethics statement

This study conformed to the tenets of the Declaration of Helsinki and was approved by the Ethics Committee of Tsukazaki Hospital. All research was performed in accordance with relevant guidelines/regulations. Written informed consent was obtained from each subject. This study was registered as UMIN000035630: ‘‘Performance comparison among different intraocular lenses in cataract surgery’’.

### Supplementary Information

Supplementary Legends.

Supplementary Table S1.

Supplementary Table S2.

Supplementary Table S3.

Supplementary Table S4.

## Data Availability

All data relevant to the study are included in this article or have been uploaded as supplementary information.
