# Supplementary material for: Comparison of visual performance between diffractive bifocal and diffractive trifocal intraocular lenses
Source: Sci Rep. 2024 Mar 4;14:5292. doi: 10.1038/s41598-024-55926-5 (PMC10912743; doi:10.1038/s41598-024-55926-5)
Supplement: Supplementary file 1 — Supplementary Legends. [file 41598_2024_55926_MOESM1_ESM.docx]

**Supplementary Information**

**Supplementary Table S1.** Patient demographics and pre-/postoperative visual parameters. For categorical data, each category and its count and frequency are shown; Fisher’s exact test (two-sided) was used to compare categorical data for the diffractive bifocal and diffractive trifocal IOLs. For numerical data, the mean and standard deviation are shown; the Mann-Whitney U test (two-sided) was used to compare numerical data for the bifocal and trifocal IOLs.

**Supplementary Table S2.** Results of multiple regression analysis of all postoperative parameters of the diffractive bifocal and diffractive trifocal groups at 10 weeks after bilateral implantation was completed. Multiple mixed linear regression or multiple linear regression was applied to numerical parameters, and cumulative logistic regression was applied to spectacle dependence parameters. In multiple linear regression and cumulative logistic regression, the variables in Table 1 were used as the explanatory variables. The regression coefficient, its 95% confidence interval, and the p-value (Wald test) are shown in (A) for each response variable. The original and corrected values (i.e., before and after adjustment with multiple linear regression) of the mean and standard deviation for each numerical parameter and the counts for each categorical parameter (Spectacle Dependence: never/sometimes/always), regression coefficient, 95% confidence interval, and p-value (Wald test) are shown in (B).

**Supplementary Table S3.** Pearson’s correlation coefficients (A) and p values from the t-tests (two-sided) of the correlation coefficients (B) of all possible combinations of postoperative parameters, which were adjusted by multiple regression with the explanatory variables in Table 1, in the bifocal group. The sample size for calculating the correlation coefficients is represented as (C).

**Supplementary Table S4.** Pearson’s correlation coefficients (A) and p values from the t-tests (two-sided) of the correlation coefficients (B) of all possible combinations of postoperative parameters, which were adjusted by multiple regression with the explanatory variables in Table 1, in the trifocal group. The sample size for calculating the correlation coefficients is represented as (C).
